# Clinicopathologic Profile, Management and Outcome of Sinonasal Ameloblastoma—A Systematic Review

**DOI:** 10.3390/jcm12010381

**Published:** 2023-01-03

**Authors:** Vini Mehta, Gargi S. Sarode, Vishnu Teja Obulareddy, Tanvi Sharma, Shruti Kokane, Marco Cicciù, Giuseppe Minervini

**Affiliations:** 1 Department of Public Health Dentistry, Dr. D.Y. Patil Dental College and Hospital, Dr. D.Y. Patil Vidyapeeth, Pune 411018, Maharashtra, India; 2 Department of Oral Pathology and Microbiology, Dr. D.Y. Patil Dental College and Hospital, Dr. D.Y. Patil Vidyapeeth, Pune 411018, Maharashtra, India; 3 S.V. Dental Clinic and Implant Centre, Hyderabad 500083, Telangana, India; 4 Department of Biomedical and Dental Sciences, Morphological and Functional Images, University of Messina, 98100 Messina, Italy; 5 Multidisciplinary Department of Medical-Surgical and Dental Specialties, University of Campania Luigi Vanvitelli, 80138 Naples, Italy

**Keywords:** ameloblastoma, paranasal sinuses, primary sinonasal ameloblastoma, paranasal sinuses, treatment, prognosis

## Abstract

Sinonasal ameloblastoma (SNA) is considered to be a subtype of ameloblastoma. It differs from gnathic ameloblastoma in terms of clinicopathologic features, management and prognosis. Thus, the objective of the present review was to study the complications, survival, recurrence rate and outcomes following the management of SNA. The electronic search process was conducted on PubMed-Medline, Embase, and Scopus. Google Scholar was used to search grey literature. Quality assessment of the case reports (CR) and case series (CS) was done based on CARE guidelines. The initial search resulted in 2111 articles. 15 studies (13 CR and 2 CS) were found to meet the eligibility criteria. The majority of the studies described histological features of SNA, which were consistent with ameloblastomas of gnathic origin. There were no SNA-related deaths reported in the included studies. Five studies described endoscopic surgeries to remove SNAs, and three SNAs were treated with post-surgery radiotherapy. Data from included studies suggest that sinonasal ameloblastomas are histologically similar to gnathic ameloblastomas, but their clinical presentation is different. They may cause complete or partial obstruction of the nasal cavity and the sinus. They appear to affect an older demographic, and their resection may be accompanied by the excision of a large portion of the maxilla, necessitating maxillofacial prosthetic rehabilitation.

## 1. Introduction

Ameloblastomas are rare tumors derived from the odontogenic epithelium [1]. More affecting the mandible than the maxilla, they are rarely malignant or metastatic and grow slowly, they can be locally invasive. Sinonasal ameloblastomas are rare tumors of the sinonasal tract that arise from sinonasal epithelium [2]. If untreated, they may lead to several complications, including tooth mobility, resorption of roots of the teeth, malocclusion and soft tissue involvement. Hence, the invasive nature of these tumors necessitates surgical resection and prosthetic rehabilitation [3]. Post-surgical rehabilitation also requires reconstructive surgery and bone grafting in addition to the construction of complex prostheses. Although less common than their mandibular counterparts, maxillary ameloblastoma are more dangerous as they may lead to the brain via the sinonasal passages and pterygomaxillary fossa [4]. The ameloblastoma originates from the remnants of dental lamina, the developing enamel organ, the epithelial lining of odontogenic cysts, or the basilar epithelial lining of epithelial cells of the gingival surface epithelium.

Histopathology of the ameloblastomas has revealed both neoplastic and cystic features [5]. They exhibit two main histological patterns: follicular or plexiform. The follicular pattern is characterized by an outer layer of columnar ameloblast-like cells surrounding an inner region of the stellate reticulum, similar to those observed in the bell stage of development of the tooth. On the other hand, a plexiform pattern is indicated by anastomosing strands (‘cord-shaped’ pattern) of epithelium with an inconspicuous stellate reticulum. Other histopathologic variants are acanthomatous, basal cell-like, granular cell and desmoplastic.

More recently, a sub-set of ameloblastomas has been described in the sinonasal tract which are so-called ‘sinonasal ameloblastomas’ (SNA) [2]. A study by Schafer et al. (1998) that surveyed 19,658 tumors associated or present in the sinonasal tract estimated that 0.11% of those tumors were ameloblastomas, making them exceedingly rare [6]. Recent literature has attempted to describe their diagnosis, treatment, and prognosis [7,8]. Untreated SNAs can lead to various complications, including rhinorrhea, nasal obstruction, and facial disfigurement due to swelling [7,8]. Progressive enlargement of SNAs can also lead to loosening and eventual loss of teeth [9]. Therefore, it is imperative to synthesize evidence regarding the clinical, radiographic and histological features, along with the treatment and diagnosis. This review aims to summarize and critically appraise the literature regarding SNAs published to date. Furthermore, we hope that the review will aid in establishing clinical guidelines for managing SNAs.

## 2. Materials and Methods

### 2.1. Focused Question

Using a modified version of the Participants, Intervention, Control and Outcomes (PICO) protocol (the Participants, Intervention and Outcomes (PIO) protocol), recommended in the Preferred Reporting Items in Systematic Reviews and Meta-Analysis (PRISMA) [10], the following focused question version was constructed: ‘What are the complication/death rates, recurrence rates, prognosis and quality of life (outcomes) reported following management (intervention) of patients with Sinonasal Ameloblastomas (participants)?’ The following types of literature were deemed eligible for inclusion: case reports (CR) and case series (CS). 

#### 2.1.1. Inclusion Criteria

- CR and CS reporting sinonasal ameloblastoma.

#### 2.1.2. Exclusion Criteria

- Gnathic ameloblastomas were excluded.
- Secondary sinonasal ameloblastomas were excluded.
- Pre-clinical studies, letters to the editor, commentaries and reviews were excluded.

### 2.2. Literature Search

The entire search process was conducted independently by two investigators. An electronic search was conducted on the following research databases: PubMed-Medline, Embase, and Scopus. Furthermore, Google Scholar was used to search grey literature (newsletters, technology assessment reports, patients and speeches) focusing on SNAs. The medical subject headings (MeSH) were: [((sinonasal ameloblastoma) OR (((sinus) OR (nasal)) AND (ameloblastoma))) AND ((treatment) OR (diagnosis) OR (prognosis) OR (recurrence) OR (oral cancer) OR (jaw lesions))]. The following journals were hand-searched: Journal of Dental Research, Oral Oncology, Journal of Cranio-Maxillo-Facial Surgery and Journal of Oral Rehabilitation. The reference lists of the included articles were scanned to find additional studies meeting our inclusion criteria. Any disagreements were solved by discussion. An inter-examiner reliability score (Kappa score) was calculated to gauge the agreeability between the examiners. Any disagreements were solved by discussion. Google Translate was used to attempt the translation of studies not in English.

### 2.3. Data Extraction

Two investigators tabulated data independently based on general criteria: ethnicity of the patients reported, country in which the study was conducted, number of participants/patients, number of SNAs reported in each study, age (mean/median or range) of the included patients, gender of the patients, features (histological, radiographic and clinical), any SNA-related deaths and follow-up. Treatment details, recurrence rate and time and any post-treatment complications were also extracted.

### 2.4. Quality Assessment

Quality assessment for CR and case series CS was done based on CARE guidelines, specifically the CARE guidelines and elaboration document [11]. Briefly, the following aspects of the studies were assessed to grade each report as ‘low’, ‘moderate’, or ‘high’: the title, keywords, quality of the abstract, introduction, reported patient information, findings of the physical/clinical examination, timeline, diagnostic assessment, reporting of interventions, follow-up details, quality of the discussion, patient perspective and informed consent/ethical approval reported.

The systematic review was registered with the International Prospective Register of Systematic Reviews on 10 November 2015, which was in accordance with the guidelines, and was last revised on 14 October 2022 (Registration Number CRD42022364686).

## 3. Results

### 3.1. Literature Search

**Figure 1:**
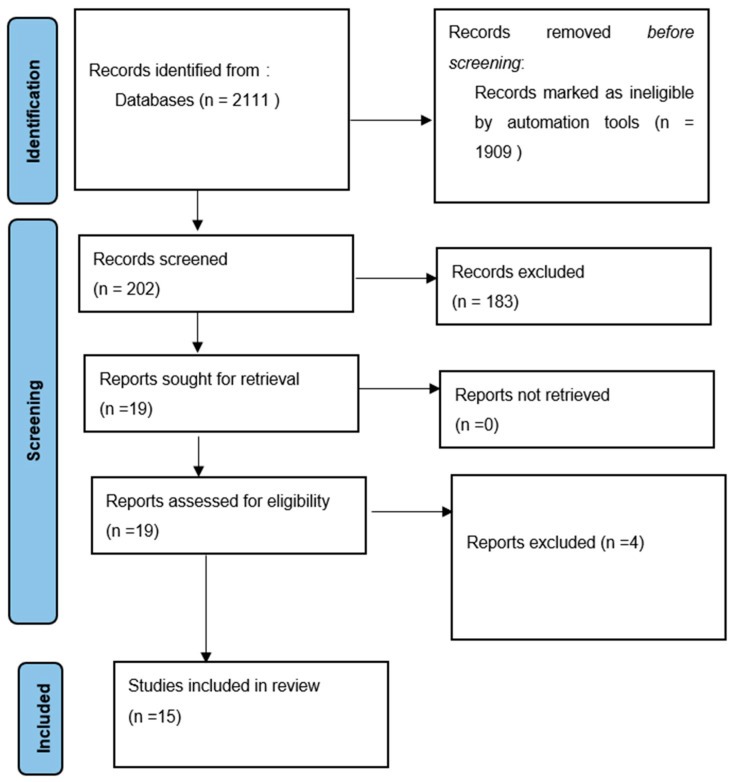
Flowchart summarizing the article selection process (n—number of studies).

The initial search resulted in 2111 articles. After the exclusion of 1909 irrelevant articles, the abstracts and titles of 202 articles were read for potential inclusion, resulting in the further exclusion of 183 articles. Therefore, the full texts of 19 articles were downloaded for potential inclusion. After the exclusion of 4 articles (one review [12], two studies that described tumors that were not SNA [13,14] and one study which could not be translated due to the limitations of Google Translate [15]), 15 studies (13 case reports [7,8,9,16,17,18,19,20,21,22,23,24,25] and 2 [6,26] case series). No additional studies were found among the references of the included studies. The Kappa score was calculated as 0.83. The literature search process is illustrated as a PRISMA flow diagram (Figure 1).

### 3.2. General Characteristics of Included Studies

The number of included patients ranged between 1 and 24 [6,7,8,9,16,17,18,19,20,21,22,23,24,25,26], and the number of SNAs in each study ranged between 1 and 24 [6,7,8,9,16,17,18,19,20,21,22,23,24,25,26]. Included studies described SNAs in 38 individuals [6,7,8,9,16,17,18,19,20,21,22,23,24,25,26]. As reported by thirteen studies, 30 of the patients were males, and 6 of them were females [6,7,8,9,16,17,19,20,21,22,23,24,25] and the age of the patients ranged between 14 and 81 years old, with the mean age at 56.90 years [6,7,8,9,16,17,18,19,20,21,22,23,24,25,26]. In one study, the age of the patient was not provided [26]. Only two studies had provided ethnicities of the patients, which were Japanese [25] and Caucasian [8]. The majority of the studies described histological features of SNA, which were consistent with ameloblastomas of gnathic origin [6,7,8,9,16,17,19,20,21,22,23,24,25].

Nevertheless, several studies also described obstruction of the nasal cavity, as revealed by computed tomography (CT) scanning or clinical examination [9,16,17,18,19,20,22]. Symptoms also included rhinorrhea [7], and nasal bleeding [17]. One study also described progressive hearing loss associated with the SNA [18]. In two studies, maxillary pain was also described [9,16]. The follow-up of patients ranged between 12 days to 44 years [6,7,8,9,16,17,18,19,21,23,24,25,26]. Three cases were reported in the USA [6,9,26], two in Germany [18,23] and Spain [16,17], and one in Australia [20], the UK [19], Iran [21], Poland [22], Japan [25], Italy [7] and China [24]. There were no SNA-related deaths reported in the included studies. The detailed general characteristics, including the clinical, histological, and radiographic features of the SNAs, are provided in Table 1.

### 3.3. Management, Recurrence Rate and Post-Op Complications

Five studies described endoscopic surgeries to remove SNAs [7,8,19,23,26] and three SNAs were treated with post-surgery radiotherapy [6,16,17]. Five studies removed the same number of SNAs with maxillectomy [6,17,20,21]. Ethmoidectomy was described in two studies [9,20] and in one study, ethmoidectomy was also stated as one of the steps for surgical management of SNA [8]. In one study, the uncinate process (a portion of the medial wall of the maxillary sinus) was resected unilaterally to allow access for the removal of the SNA [24]. In two studies, the recurrence (or absence of recurrence) was not reported [9,26]. Three studies reported a post-surgical recurrence at 6 months, 10 months and 2 years [8,24,25]. One study reported a 5% recurrence rate within 1 to 13 years after surgery of 24 SNAs [6]. In one study, tooth 27 was extracted because it had developed numbness post-surgery [8]. In one study, the recurrent mass was not removed [25]. A detailed description is given in Table 2.

### 3.4. Results of the Quality Assessment

In seven studies (47%), the type of the study was stated in the title [6,7,19,20,22,24,25], and in none of the studies, ‘CR’ or ‘CS’, was used as a keyword. In eleven studies, the background was provided adequately in the abstracts [6,7,8,9,16,17,19,22,23,24,25]. The main findings in the abstract were reported adequately in twelve studies [6,7,8,16,17,19,21,22,23,24,25,26]. Conclusion in the abstract was provided in twelve studies [6,7,8,16,17,18,19,21,22,23,24,25]. An adequate introduction was provided in six studies [6,8,16,20,21,22]. De-identified patient information was provided in fourteen studies [6,7,8,9,16,17,18,19,20,21,22,23,24,25]. The main concerns and symptoms of the patients were reported in eleven studies [6,9,16,17,18,19,20,21,22,23,24]. Medical, family, psychosocial and genetic history was provided adequately in three studies [18,21,24], and partially in one study [25]. A history of past interventions was provided in five studies [8,21,22,24,25]. In thirteen case reports, adequate physical examination description was provided [6,7,8,9,16,17,18,20,21,22,23,24,26]. None of the studies included a timeline of the treatment of the included patient(s). Diagnostic testing was carried out adequately in thirteen studies [6,7,8,9,16,17,18,20,21,22,23,24,26] and partially in one study [25]. None of the studies described the practitioners facing challenges during diagnostic testing, but all provided an adequate diagnosis of the patient(s) treated [6,7,8,9,16,17,18,19,20,21,22,23,24,25,26]. Adequate prognostic or staging information was provided in eleven studies [6,7,8,9,16,18,21,22,23,24,26]. While the type of surgical or radiotherapeutic information was provided sufficiently in all studies [6,7,8,9,16,17,18,19,20,21,22,23,24,25,26], seven studies did not provide details or level of the surgical procedure and/or the dose of radiation to which the patient was exposed [7,16,17,19,20,21,25].

Furthermore, none of the CR suggested that the practitioners had deviated from their original treatment plan. Patient- or clinician-reported outcomes were described in eleven studies [6,7,8,9,16,17,21,22,23,24,26]. An adequate description of follow-up testing was provided in five studies [8,16,18,23,24]. Adherence and tolerability to follow-up of the patients were described in three studies [6,24,26]. Post-op complications (adverse effects) were described in only one study [27]. When the discussion section of the studies was assessed, limitations and weaknesses were described in one study [21]. The relevant literature was discussed adequately in thirteen studies [6,7,8,9,16,17,19,20,21,22,24,25,26] and partially in two of them [18,23]. In ten case reports, the conclusion was justified satisfactorily [6,8,16,17,20,22,23,24,25,26]. Recommendations or ‘take-away’ lessons were provided in nine case studies [6,8,9,16,18,20,22,24,25,26]. Patient perspectives were described in one study [6] and ethical information or consent was provided in just two studies. [9,24] Therefore, as presented in Table 3, eleven studies were graded as having ‘low’ quality [7,9,16,17,18,19,20,21,23,25,26], two studies were graded as ‘moderate’ [6,8] and two other studies were graded as ‘high’ [22,24]. A detailed description is given in Table 3.

## 4. Discussion

Sinonasal ameloblastomas are a relatively recent sub-type of maxillary ameloblastomas. Given this, to the best of the authors’ knowledge, this systematic review is the first such paper that has summarized the overall outcomes of the management of SNAs and the features of the tumors. Overall, data from 38 individuals (and the same number of SNAs) within the included studies suggest that SNAs may have a recurrence of approximately 21% [6,7,8,9,16,17,18,19,20,21,22,23,24,25,26], which is slightly lower than the recurrence rate of ameloblastomas in general (23.5%) [28]. Most studies have described the histological features of SNAs as similar to those of gnathic ameloblastomas [6,7,8,9,16,17,18,19,20,21,22,23,24,25,26] summarized in Table 1. The follicular is the most common histopathologic pattern seen in gnathic ameloblastomas, while plexiform was found to be the most common pattern in SNA [6]. Immunohistochemical markers such as cytokeratins 5/6, 13, 14 and 19 are used for confirming the diagnosis of gnathic as well as SNAs [25].

Nonetheless, the most striking difference is the clinical presentation of SNAs compared to conventional maxillary ameloblastomas. Maxillary ameloblastomas have been observed to expand more rapidly than their mandibular counterpart due to a thinner bone of the maxilla, making them more likely to invade the brain [29]. Additionally, SNAs several studies have described the tumors causing nasal obstruction, sinusitis, and rhinorrhea [9,16,17,18,19,20,22], a clinical presentation that may help distinguish between SNAs from other types of maxillary ameloblastomas. The mean age of the patients at which SNAs were diagnosed was approximately 60 years [6,7,8,9,16,17,18,19,20,21,22,23,24,25,26], which is considerably higher than 30–40 years reported in previous studies [30,31].

Several reports required maxillectomy to remove SNAs and the structures they had invaded [6,17,20,21]. To date, no guidelines have been established for the optimal surgical management of SNAs. Still, due to their locally invasive nature, prosthodontic or maxillofacial prostheses are very likely to be needed to effectively rehabilitate such patients. In none of the studies a comprehensive rehabilitation treatment plan was described. In one study, post-surgical paresthesia of the upper second molar was reported [8], which could be most likely due to trauma to a sensory nerve to the tooth.

Overall, the quality of the studies included in this review was low. The majority of the studies did not report the ethnicities of the affected patients. Similarly, to date, no study has established a racial predilection to ameloblastoma. Furthermore, most studies have described detailed surgical procedures for removing SNAs, which would be crucial for future guidelines.

Although no SNA-related deaths were reported, the included patients’ sample size was insufficient to ascertain the survival statistics. The recurrence rate of ameloblastoma depends on many factors such as histological variant, site and the management. It is more frequent in mandible than maxilla. Follicular ameloblastoma has a higher recurrence rate compared to plexifom. Thus, the site and the histologic variant could be the reasons for the lower recurrence rate of SNAs compared to gnathic ones. Cases treated with a conservative approach show a significantly higher recurrence rate compared to the cases treated with a radical approach. Recurrence was reported only in four studies which included nine cases. Out of the total nine cases, five cases recurred in a span of 1 to 13 years, two after 2 years and two within 6 to 10 months. There are no complications reported in any of the studies, except one which mentioned numbness associated with a tooth.

This systematic review has some limitations. Firstly, due to the nature of the pathology studied, all the studies were either case reports or series—which may have several sources of bias. Furthermore, data from only 38 cases were included. In addition, the classification of SNAs based on the type, i.e., unicystic or solid multicystic could not be taken into consideration. Additionally, it was not feasible to carry out a meta-analysis due to the nature of the studies included.

## 5. Conclusions

Data from included studies suggest that SNAs are histologically similar to gnathic ameloblastomas, but their clinical presentation is different. They may cause complete or partial obstruction of the nasal cavity and the sinus. Additionally, they may also lead to rhinorrhea and nasal bleeding. Furthermore, they appear to affect an older demographic (mean age 59 years), and their resection may be accompanied by the excision of a large portion of the maxilla, necessitating maxillofacial prosthetic rehabilitation. SNAs have a better outcome in terms of recurrence and complications, however the histopathologic variant and management approach should be taken into consideration. Nevertheless, more cases should be reported adequately so that guidelines may be developed for diagnosing and managing SNAs to have a better outcome.

## Figures and Tables

**Table 1:** Overview of included studies.

Study (Author(s), Year)	Country	Ethnicity	Participants/Cases with SNA (n)	SNA (n)	Age (Mean/Median, Range; Years)	Gender (n)	Features/Presentation			Deaths Due to Ameloblastoma	Follow-up (Mean & Range, Years)
							Histological	Radiographic	Clinical		
Schafer et al., 1998 [6]	USA	NA	24	24	Mean 59.7; Range: 43–81 years	M: 19; F: 5	Peripheral palisaded columnar cells with reverse polarity	Solid masses or opacities in nasal cavity, maxillary sinus, or both Plexiform pattern Surface epithelial derivation	Enlarging mass (n = 24) Sinusitis (n = 9) Epistaxis (n = 8)	0	9.5; 1–44
London et al., 2002 [26]	USA	NA	1 (out of 18)	1	NR	NR	NR	NR	NR	0	4 years
Guilemany et al., 2004 [16]	Spain	NA	1	1	68 years	M: 1	Long anastomosing strands of odontogenic epithelium	Bony erosion of lateral sinus and orbital floor	Headache, maxillary pain, facial paresthesia, nasal obstruction, rhinorrhea	0	50 months
Ereno et al., 2005 [17]	Spain	NA	1	1	66 years	M: 1	Anastomosing epithelial cords within a hyaline fibrous stroma; arising from surface epithelium of maxillary sinus	Opaque mass in the nasal cavity, and maxillary and ethmoidal sinuses with loss of integrity of alveolar area	Nasal obstruction and bleeding	0	9 months
Koscielny et al., 2020 [18]	Germany	NA	1	1	56 years	NR	Epithelial-mesenchymal tumor, mimicking primitive tooth formation	Mass obstructing right nasal cavity.	Progressive hearing loss and nasal obstruction for one year	0	1 year
Leong et al., 2010 [19]	UK	NA	1	1	61 years	M: 1	Respiratory mucosa infiltrated by interconnecting strands and cords of epithelium in loose, vascular sparsely cellular connective tissue stroma indicating	Complete obstruction of nasal cavity with deviated nasal septum to the other side. Middle turbinate obliterated.	Nasal obstruction, blood-stained mucus. Lesion: lobulated, vascular.	0	12 months
Morrisson et al., 2011 [20]	Australia	NA	1	1	73 years	F: 1	Follicular pattern; cords of tumor within a hyalinized fossa	Expansive, erosive mass obstructing nasal cavity and nasopharyngeal air space	Complete nasal obstruction; polypoid mass.	0	NR
Shahidi et al., 2012 [21]	Iran	NA	1	1	74 years	M: 1	Follicular islands of odontogenic epithelium presenting follicular ameloblastoma	Erosion of alveolar process and left premolar and molar region. Loss of borders of maxillary sinus and lateral wall of nasal fossa. Massive expansile lesion invading the entire affected maxillary sinus	Yellowish-white necrotized tissue surrounded by an erythemic rim and mucosal hyperplasia. Bony swelling in the premolar and molar region	0	4 years
Temporale et al., 2013 [22]	Poland	NA	1	1	35 years	M: 1	Ameloblastoma	Soft tissue mass involving nasopharynx, ethmoid and sphenoid sinus	Nasal obstruction occurred 2 months after removal of nasal cyst noted after septoplasty	0	NR
Barrena et al., 2019 [9]	USA	NA	1	1	34 years	M: 1	Ameloblastic islands: hyperchromatic columnar cells with reverse polarity.	Pain and selling in left maxillary for 2–3 weeks	Well-demarcated, soft tissue mass in left maxillofacial region. Complete obstruction of left maxillary sinus, nasal cavity, and ethmoid sinus. Extension to alveolar process, body of zygoma and floor of orbit. Involvement as far as posterior portion of body of sphenoid.	0	12 days
Harada et al., 2020 [25]	Japan	Japanese	1	1	80 years	M: 1	Basaloid cells with cystic structures; columnar or cuboidal epithelial cells.	Mass initially diagnosed as a polyp and misdiagnosed as a salivary gland tumor.	MRI revealed lesion in right nasal cavity and right maxillary sinus.	0	2 years
Fiedler et al., 2021 [23]	Germany	NA	1	1	38 years	M: 1	NA	Nasal obstruction	Completely obstructed right maxillary sinus and distortion of middle and inferior conchae.	0	4 months
Karp et al., 2021 [8]	Not clear	Caucasian	1	1	64 years	M: 1	Cyst-like; columnar epithelial cells.	Inferomedially to the root of the upper left third molar	Diagnosis of SNA made during repeat endoscopic sinus surgery.	0	15 months
Tranchina et al., 2021 [7]	Italy	NA	1	1	74 years	M: 1	Cords and follicular islands of odontogenic epithelium; columnar cells with reverse polarity.	Lytic, expansile, solid lesion; from nasopharynx to lateral pharyngeal space, laterally to the parotid. Erosion of bone in the middle cranial fossa	2 months of progressive right-side obstruction, rhinorrhea and sinusitis	0	12 months
Wu et al., 2022 [24]	China	NA	1	1	14 years	M: 1	Capsule wall-like substance composed of fibrous tissue lined with odontogenic epithelium and epithelial nests and calcium deposits	Expansive bone destruction of the maxillary sinus	Swelling noted on left cheek one month after a cold	0	16 months

NA: Not Applicable; SNA: Sinonasal ameloblastoma; M: Male; F: Female, n: number, MRI: Magnetic resonance imaging.

**Table 2:** Treatment, recurrence rate and post-op complications of included studies.

Study (Author(s), Year)	Treatment	Recurrence (n (%), Time Post Treatment)	Post Treatment Complications
Schafer et al., 1998 [6]	Surgical excision (n = 23) Maxillectomy + radiotherapy (n = 1)	5 (21%), 1–13 years	None
London et al., 2002 [26]	Computer assisted endoscopy	NR	None
Guilemany et al., 2004 [16]	Resection; paralateral rhinotomy; radiotherapy	0	None
Ereno et al., 2005 [17]	Radical right maxillectomy with radiotherapy	0	None
Koscielny et al., 2020 [18]	Maxillary resection through lateral rhinotomy	0	None
Leong et al., 2010 [19]	Endoscopic resection	0	None
Morrisson et al., 2011 [20]	Right total maxillectomy and ethmoidectomy, with clearance of right infratemporal fossa. Reconstruction with a vertical rectus abdominis mycocutaneous flap	0	None
Shahidi et al., 2012 [21]	Radical left maxillectomy	0	None
Temporale et al., 2013 [22]	Radical surgery—access through eversion of face coverings (removal of front and medial call of right maxillary sinus wall	0	None
Barrena et al., 2019 [9]	Unilateral total ethmoidectomy, frontal sinusotomy, sphenoid sinusotomy with left infratemporal dissection. Free flap reconstruction and orbital reconstruction	NR	None
Harada et al., 2020 [25]	Surgical excision	After 2 years	Not available–recurrent mass left untreated
Fiedler et al., 2021 [23]	Transnasal functional endoscopic sinus surgery	0	None
Karp et al., 2021 [8]	Endoscopic (transnasal) turbinectomy and medical maxillectomy	1 (6 months post-op)	Numbness of tooth 27—extracted.
Tranchina et al., 2021 [7]	Endoscopic excision	0	0
Wu et al., 2022 [24]	Left uncinate process resected endoscopically and maxillary sinus was opened for access (two surgeries required due to recurrence)	1 (10 months)	None

**Table 3:** The results of the quality assessment of the included studies using the CARE guidelines checklist.

Topic	Schafer et al., 1998 [6]	London et al., 2002 [26]	Guilemany et al., 2004 [16]	Ereno et al., 2005 [17]	Koscielny et al., 2020 [18]	Leong et al., 2010 [19]	Morrisson et al., 2011 [20]	Shahidi et al., 2012 [21]	Temporale et al., 2013 [22]	Barrena et al., 2019 [9]	Harada et al., 2020 [25]	Karp et al., 2021 [8]	Fiedler et al., 2021 [23]	Tranchina et al., 2021 [7]	Wu et al., 2022 [24]
Title (mentioning case report)	Yes	No	No	No	No	Yes	Yes	No	Yes	No	Yes	No	No	Yes	Yes
Key words (with ‘case report’)	No	No	No	No	No	No	No	No	No	No	No	No	No	No	No
Abstract															
Background	Yes	No	Yes	Yes	No	Yes	No	No	Yes	Yes	Yes	Yes	Yes	Yes	Yes
Main findings	Yes	Yes	Yes	Yes	No	Yes	No	Yes	Yes	No	Yes	Yes	Yes	Yes	Yes
Conclusion	Yes	Yes	Yes	Yes	No	Yes	No	No	Yes	No	Yes	Yes	Yes	Yes	Yes
Adequate introduction	Yes	No	Yes	No	No	No	Yes	Yes	Yes	No	No	Yes	No	No	No
Patient information															
De-identified information	Yes	No	Yes	Yes	Yes	Yes	Yes	Yes	Yes	Yes	Yes	Yes	Yes	Yes	Yes
Concerns and symptoms	Yes	No	Yes	Yes	Yes	Yes	Yes	Yes	Yes	Yes	No	No	Yes	No	Yes
Medical, family, psychosocial genetic history	No	No	No	No	Yes	No	No	Yes	No	No	Partially	No	No	No	Yes
Past interventions and outcomes	No	No	No	No	No	No	No	Yes	Yes	No	Yes	Yes	No	No	Yes
Physical examination and clinical findings	Yes	Yes	Yes	Yes	Yes	Yes	No	Yes	Yes	Yes	No	Yes	Yes	Yes	Yes
Timeline	No	No	No	No	No	No	No	No	No	No	No	No	No	No	No
Diagnostic assessment															
Testing	Yes	Yes	Yes	Yes	Yes	No	Yes	Yes	Yes	Yes	Partial	Yes	Yes	Yes	Yes
Challenges	No	No	No	No	No	No	No	No	No	No	No	No	No	No	No
Diagnosis	Yes	Yes	Yes	Yes	Yes	Yes	Yes	Yes	Yes	Yes	Yes	Yes	Yes	Yes	Yes
Prognosis/staging	Yes	Yes	Yes	Yes	No	No	No	Yes	Yes	Yes	No	Yes	Yes	Yes	Yes
Intervention															
Type of intervention stated	Yes	Yes	Yes	Yes	Yes	Yes	Yes	Yes	Yes	Yes	Yes	Yes	Yes	Yes	Yes
Dosage/level/details of intervention	Yes	Yes	No	No	Yes	No	No	No	Yes	Yes	No	Yes	Yes	No	Yes
Changes/modifications	No	No	Yes	No	No	No	No	No	No	No	No	No	No	No	Yes
Follow-up/outcome details reported															
Clinician-/patient-reported outcomes	Yes	Yes	Yes	Yes	No	No	No	Yes	Yes	Yes	No	Yes	Yes	Yes	Yes
Follow-up diagnostic tests	No	No	Yes	No	Yes	No	No	No	No	No	Yes	Yes	Yes	No	Yes
Adherence and tolerability to follow-up tests	Yes	Yes	No	No	No	No	No	No	No	No	No	No	No	No	Yes
Adverse effects reported	No	No	No	No	No	No	No	No	No	No	No	Yes	No	No	No
Discussion															
Strengths and limitations	No	No	No	No	No	No	No	Yes	No	No	No	No	No	No	No
Discussion of relevant literature	Yes	Yes	Yes	Yes	Partially	No	No	Yes	Yes	Yes	Yes	Yes	Partially	Yes	Yes
Rationale for conclusions	Yes	Yes	Yes	Yes	No	No	No	No	Yes	No	Yes	Yes	Yes	No	Yes
Take-away lessons/recommendations	Yes	Yes	Yes	No	Yes	No	No	No	Yes	No	Yes	Yes	No	No	Yes
Patient perspective	Yes	No	No	No	No	No	No	No	Yes	No	No	No	No	No	No
Informed consent/ethical approval	No	No	No	No	No	No	No	No	No	Yes	No	No	No	No	Yes
Overall quality	Moderate	Low	Low	Low	Low	Low	Low	Low	High	Low	Low	Moderate	Low	Low	High

## Data Availability

Not applicable.
